# Comparison of the Impacts of Under-Treated Pain and Opioid Pain Medication on Cognitive Impairment

**DOI:** 10.7759/cureus.22037

**Published:** 2022-02-08

**Authors:** Sung Eun Jang, Ylisabyth S Bradshaw, Daniel B Carr

**Affiliations:** 1 Family Medicine, Elliot Health System, Bedford, USA; 2 Public Health and Community Medicine, Tufts University School of Medicine, Boston, USA

**Keywords:** systematic review, cognitive function, memory, opioid, pain

## Abstract

Purpose: To guide clinicians in balancing the risks and benefits of opioids when treating pain, we conducted two systematic reviews: 1) the impact of pain on cognitive function, and 2) the impact of opioids on cognitive function.

Methods: Part one addressed the impact of pain on cognitive impairment; Part two considered the impact of opioids on cognitive impairment. PubMed was used to search for eligible articles. For part one, 1786 articles were identified, of which 23 met our eligibility criteria. For part two, among 584 articles, 18 were found eligible.

Results: For part one, 16 studies concluded that patients with chronic pain showed impaired cognitive function; six studies found that chronic pain does not worsen cognitive function; one study concluded that the impact of pain on cognitive function differs based on the underlying cognitive status. For part two, 15 studies found that using opioids to control pain did not cause significant cognitive impairment, while three studies concluded the opposite. Studies evaluating older subjects did not observe different results from those in the whole population for both reviews.

Conclusion: The published literature indicates that moderate to severe pain can impair cognitive function, and that careful use of opioid analgesics in such subjects does not necessarily worsen cognition. Although our results are insufficient to support clear guidance due to heterogeneity of cohorts and outcomes, this study may assist primary care providers by rendering explicitly the factors to be considered by providers caring for this population with pain when opioids are considered.

## Introduction and background

The opioid crisis is a nationwide concern. Misused opioids can lead to overuse, substance use disorder, and serious health outcomes. However, the use of opioids is frequently considered when other treatment modalities fail to manage pain [1].

One of the main concerns related to opioids is cognitive impairment [2], especially, for the frail elderly population [3]. Ironically, undertreated pain itself may increase the risk of cognitive impairment [4].

Currently, recommendations to guide the judicious use of opioids in this context are limited. Beers Criteria [5] (potentially inappropriate medication use guideline from the American Geriatric Society) state that opioid analgesics can cause ataxia, impaired psychomotor function, syncope, and additional falls. It recommends providers to avoid using opioids except for pain management in the setting of severe acute pain, such as recent fractures or joint replacement. As this is a rather general statement, it is desirable to have a more practical, evidence-based recommendation.

Previous literature focuses on either the effect of opioids on cognitive impairment or the effect of undertreatment of pain on cognitive impairment, respectively. A comprehensive review of risk assessment on both aspects is conducted in the present systematic review.

## Review

Methods

This review project consists of two parts. Part one is a systematic review of the impact of pain on cognitive function. For this part of the review, the study group was identified as patients with pain and the control group was participants with no pain. Part two is a systematic review of the impact of opioids on cognitive function. For part two, the study group was patients being treated opioids alone or in combination with other analgesic drugs whereas the control group was people with no treatment or analgesics other than opioids.

For both parts, the principal outcome of interest was cognitive impairment.

Inclusion criteria

Age inclusion criteria were set for age 19 or older, with no upper limit. Subgroup analysis for the age group over 65 years old was performed. All opioid formulations, including oral, intravenous, sublingual, and transdermal, were included in this review.

Exclusion criteria

Articles based on self-reported cognitive impairment were excluded. Cancer pain or pain in palliative care was not considered in our review. Articles focusing on the outcome of neuropsychological conditions, such as delirium, agitation, were excluded, as we focused on long-term impairment of cognitive function, rather than transient or short-term complications. Review, systematic review, or meta-analysis articles were excluded. Studies not available in English full text were also excluded.

Search strategy

We used Pubmed to search for studies published up to December 2020, as it appears in Table 1. No difference in retrieved articles was found using Medline search. 

**Table 1:** Search term details.

Review part	Search term on Pubmed
Pain and cognitive impairment	((((("persistent pain" OR "chronic pain" OR "chronic non-cancer pain" OR "chronic non-cancer pain" OR "chronic non-cancer pain" OR "chronic non-malignant pain" OR "chronic neuropathic pain")) AND ("cognitive function*" OR "cognitive impairment" OR cognition OR "neuropsychological test*" OR neuropsychology OR driving OR "Neuropsychological Tests"[Mesh] OR memory loss OR MOCA OR MMSE)) AND Humans[Mesh])) AND (cohort OR clinical trial OR randomized controlled trial OR cross sectional OR case control)
Opioid and cognitive impairment	((((((opiate OR opioid) OR (morphine OR methadone OR fentanyl OR codeine OR buprenorphine OR tramadol OR tapentadol OR oxycontin OR hydromorphone OR hydrocodone) OR ("Analgesics, Opioid"[Mesh]))) AND (("persistent pain" OR "chronic pain" OR "chronic non-cancer pain" OR "chronic non-cancer pain" OR "chronic non-cancer pain" OR "chronic non-malignant pain" OR "chronic non-malignant pain" OR "chronic non-malignant pain" OR "chronic neuropathic pain"))) AND (("cognitive function*" OR "cognitive impairment" OR cognition OR "neuropsychological test*" OR neuropsychology OR driving OR "Neuropsychological Tests"[Mesh])))) AND Humans[Mesh]

Study selection process

Pain and Cognitive Impairment (Part One)

**Figure 1:**
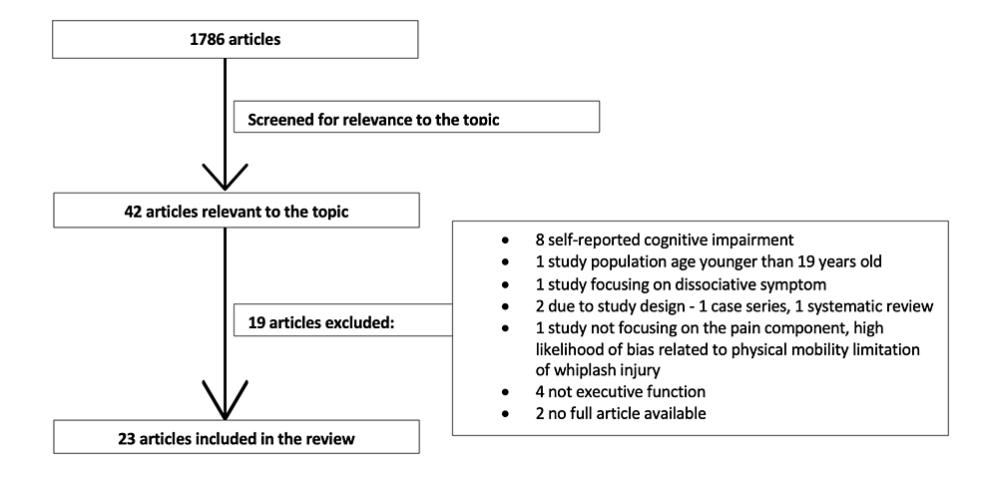
Literature search: impact of pain on cognitive impairment.

We found 1786 articles using the Pubmed search engine, as it appears in Figure 1. Forty-two were identified as being relevant to the topic. Nineteen of the 40 articles were excluded for the following reasons. Eight articles used self-reported cognitive impairment. One focused on the study population younger than 19 years old. One studied dissociative symptom. Two were excluded due to study design, being a case series and a systematic review. One study was excluded due to high likelihood of bias, as it evaluated the effect of whiplash associated disorder (WAD) on driving function, which is more likely related to the physical mobility limitation of whiplash injury than pain. Four studies were excluded as they focused on neuropsychological conditions such as delirium. Two were excluded due to lack of the full text. Some 23 articles [6-28] were included in the final review.

Opioids and Cognitive Impairment (Part Two)

**Figure 2:**
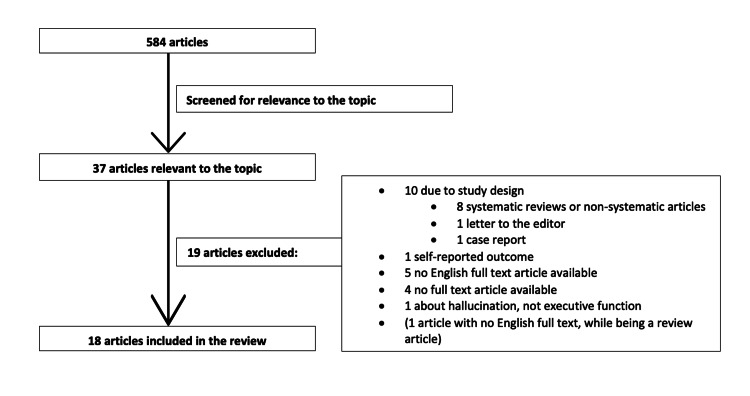
Literature search: opioids and cognitive impairment.

We found 584 articles in our Pubmed search, as it appears in Figure 2. After screening for the relevance to the topic, 37 articles remained. Among those, 19 articles were excluded for the following reasons. Eight studies were review articles, one was a letter to the editor, and one was a case report. Five articles were not available with English full text. Four studies did not have any full text. One study was excluded as it considered the outcome of hallucination. Some 18 articles [29-46] were included in the final review.

Results and analysis

Part One: Impact of Pain on Cognitive Impairment

Demographic information 

Demographic information is described in Table 2. Out of the 23 studies being reviewed, eight studied the geriatric population over 65 years old. Three studies did not have control group, and three studies had multiple study subgroups. In general, more females were studied than males.

**Table 2:** Demographic information: impact of pain on cognitive impairment.

Study sorted by author name	Number of participants (study group/control)	Age (years old) (study group/control)	Gender (study group male/female; control group male/female)
Baarbe et al. [6]	13 /12	21.2 ± 1.9 years; 21.9 ± 2.1 years	5/8;9/3
Coppieters et al. [7]	59 participants [16 chronic whiplash-associated disorder patients, 21 fibromyalgia (FM) patients, and 22 pain-free volunteers]	WAD 41.62; FM 44.52; Control 38.00	WAD 3/13; FM 5/16; Control 8/14
Ferreira Kdos et al. [8]	45 patients with chronic pain and on 45 control subjects	46.9; 45.1	11/34; 11/34
Harman et al. [9]	40 patients (20 patients with persistent pain and 20 controls)	40.4; 44.6	4/16; 3/17
Ickmans et al. [10]	27 chronic WAD and 27 controls	41.4 +/10.8; 41.7+/- 14.7	11/16; 11/16
Ickmans et al. [11]	29 female chronic fatigue syndrome (CFS) patients, 17 healthy controls	35.4; 35.6	All females
Jorge et al. [12]	23 RA, 21 chronic low back pain (CLBP)	Rheumatoid arthritis (RA) 57.4; cLBP 52.66;	RA 3/20; cLBP 5/16
Ling et al. [13]	50 participants with chronic pain, 50 pain-free participants	52.14; 49	23/27; 22/28
Liu et al. [14]	176 outpatients with chronic pain and 170 health controls	34.84; 33.91	88/88; 89/81
Masiliunas et al. [15]	29 patients with cLBP, 30 healthy volunteers	59.6; 60.7	13/16; 14/16
Murata et al. [16]	44 chronic musculoskeletal pain group, 190 control group	74.6; 72.2	10/34; 77/113
Ren et al. [17]	24 somatoform pain disorder (SPD), 24 control	36.5; 33.2	12/12; 13/11
Scherder et al. [18]	19 patients in an early stage of probable AD, 20 older persons without dementia	Alzheimer's disease (AD) 86.37; Without dementia 85.70	AD 2/17; Without dementia 4/16
Schiltenwolf et al. [19]	33 cLBP, 25 healthy controls	49.82 (28–71); 45.88 (35–66)	8/25; 15/10
Schmand et al. [20]	65 Whiplash non-malingering, 43 Whiplash malingering, 20 Closed head injury, 46 controls	Whiplash non-malingering 37.2; Whiplash malingering 41.0; Closed head injury 37.7; Control 33.9	Whiplash non-malingering 31/34; Whiplash malingering 14/29; Closed head injury 12/8; Control 17/29
Schuler et al. [21]	55 chronic pain, 36 acute pain	Chronic pain 80.8; Acute pain 81.4	Chronic pain 12/43; Acute pain 6/30
Sjogren et al. [22]	91 chronic nonmalignant pain patients, (21 with no pain medications, 19 in long-term oral opioid treatment, 18 treated with antidepressants and/or anticonvulsants, 33 treated with long-term oral opioids and antidepressants and/or anticonvulsants) 64 controls	40.4; 46.4; 40.3; 48.1; 47.6	5/16; 11/8; 6/12; 17/16; 29/35
Terassi et al. [23]	187 elderly caregivers with chronic pain, 133 without chronic pain	67 (60-95); 69 (60-98)	36/151; 41/92
Tomey et al. [24]	1413 participants	53	All females
van der Leeuw et al. [25]	765 participants in MOBILIZE study	Average age of 78.1 years	276/489
Weiner et al. [26]	160 cLBP, 163 pain-free patients	73.6; 73.5	83/80; 94/66
Whitlock et al. [27]	10065 community-dwelling older adults. 1120 persistent pain, 8945 controls	73.8; 73.6	269/851; 3748/5197
Zanocchi et al. [28]	105 patients	82.2 +/- 9.0	31/74

Study design

Fourteen out of 23 studies reviewed were cross-sectional studies. The remainder were four prospective cohort studies, three case-control studies, and two retrospective cohort studies.

Pain type, chronicity, and intensity

Musculoskeletal pain was the most studied. Nine articles studied musculoskeletal pain such as neck, back, or mixed pain. Three studied WAD. Two studied mixed type of pain, one focused on chronic fatigue syndrome (CFS), and one focused on somatoform pain disorder (SPD). Eight studies did not report the type of pain included.

Most of the reviewed articles studied chronic pain except for one article that included patients with acute or chronic pain.

Moderate intensity pain was most commonly studied. Eight articles included patients with moderate pain. Three studied moderate to severe pain, two studied mild pain, and one focused on severe pain. Six articles did not report pain intensity.

Cognitive testing

**Figure 3:**
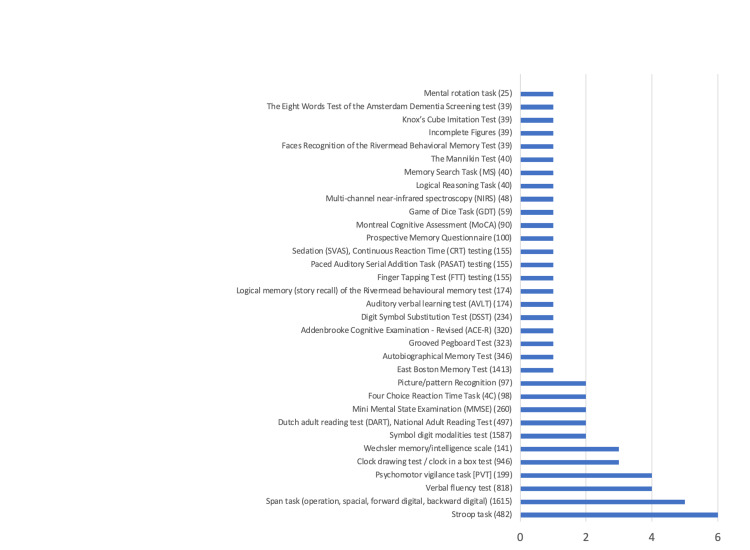
Numbers of studies using specific cognitive tests (number of participants): impact of pain on cognitive impairment.

Various types of cognitive testing modalities were used in the reviewed studies, as it appears in Figure 3. Some 33 cognitive tests were used in 23 studies. This diversity of the tests being used to assess cognitive function makes it challenging to combine their reported results. The Stroop task was used most often, followed by the Span task and the verbal fluency test. Tests that are commonly used in clinical practice, such as Mini Mental State Examination (MMSE) (two studies) or Montreal Cognitive Assessment (MOCA) (one study), were used infrequently in the reviewed research.

Results

Sixteen out of 23 reviewed studies concluded that patients with chronic pain have worse cognitive function than patients without pain. Six studies presented opposite findings, i.e. that chronic pain does not worsen cognitive function. Also, one study [18] stated that the impact of pain on cognitive function differs based on the underlying cognitive impairment. In this study, the Alzheimer’s group showed a significant correlation between pain and cognitive decline; however, the cognitively intact group did not show a significant correlation between those two.

The studies concluding that pain does not impair cognitive function enrolled a relatively small number of participants, as it appears in Table 3. Confounding factors such as underlying cognitive impairment, malingering, and level of education or intelligence were not adequately addressed. The importance of such confounding factors is attested to one study [18], which concluded that the relationship between pain and cognitive impairment differs based on the underlying cognitive function level. Also, one study [10] had a short follow-up time, which makes it hard to predict long-term effects. Regarding study design, they were mostly (61%) cross-sectional studies.

**Table 3:** Studies with the conclusion that pain does not impair cognitive function: impact of pain on cognitive impairment.

Study sorted by author name	Number of participants (study group/control)	Age (years old) (study group/control)	Pain type	Pain intensity	Study design	Results	Author-identified limitations
Ickmans et al. [10]	27 chronic whiplash-associated disorders (WAD) and 27 controls	41.4 +/10.8; 41.7+/- 14.7	Chronic WAD	Moderate	Prospective cohort	In the short term, post-exercise cognitive functioning, pain, and fatigue were not aggravated in people with chronic WAD	The longer-term effects of exercise (therapy) on cognitive functioning in people with chronic WAD need to be studied.
Ickmans et al. [11]	29 female chronic fatigue syndrome patients, 17 healthy controls	35.4; 35.6	Pain with chronic fatigue syndrome	Not available (N/A)	Retrospective cohort	Pain severity was not associated with cognitive performance	Large number of correlations. Only women. Malingering, education, intelligence not studied. Causal relationship is possible.
Masiliunas et al. [15]	29 patients with chronic lower back pain (cLBP), 30 healthy volunteers	34.84; 33.91	cLBP	Moderate (current pain in visual analog scale, VAS 56)	Case control	cLBP patients did not score significantly worse in any examined neuropsychological tests.	Small sample size control group mild cognitive impairment (MCI) not excluded. Possible low motivation and effort to succeed. Trail Making Test, subjects were asked not only to connect the dots but also to encircle them.
Schuler et al. [21]	55 chronic pain, 36 acute pain	Chronic pain 80.8; Acute pain 81.4	Chronic and acute pain	Moderate	Cross sectional	Report of pain intensity and improvement in the activities of daily living (ADL) measure was independent of cognitive status	Small sample size
Terassi et al. [23]	187 elderly caregivers with chronic pain, 133 without chronic pain	67 (60 - 95); 69 (60 - 98)	Chronic pain	Moderate (39.0%), Severe (38.6%) (Mean 6.41)	Cross sectional	No difference was found in cognitive performance among the elderly with chronic pain and those without chronic pain for any domain of Addenbrooke's Cognitive Examination Revised (ACE-R) instrument.	No use of neuroimaging techniques or specific tests for memory, attention, and concentration No information about the duration of chronic pain. No controlled variables (meds, depression, sleep disorders)
Zanocchi et al. [28]	105 patients	82.2 +/- 9.0	Chronic pain	N/A	Cross sectional	In institutionalized elderly, there was no correlation between chronic pain and cognitive function change	Small sample size. Difficulty removing confounding factor (age and sex). Difficulty assessing pain in patients with cognitive impairment. Many sample patients with moderate cognitive impairment.
Scherder et al. [18]	19 patients in an early stage of probable AD, 20 older persons without dementia	AD 86.37; Without dementia 85.70	Arthrosis/arthritis	Moderate	Cross sectional	In cognitively intact older persons, there is no significant correlation between specific cognitive functions and pain intensity/effect. In the Alzheimer's dementia (AD) group, there is a significant positive correlation.	The present findings can only be generalized to older adults who do not suffer from a major depressive disorder

Age was not a factor influencing these different results. Four out of those seven studies, with the conclusion that pain does not worsen cognitive function, studied the geriatric population.

 Studies focusing on ages over 65 years old 

As described in Table 4, all eight articles studying the geriatric population focused on chronic pain, and musculoskeletal pain was studied in three of these eight. Seven articles were cross-sectional studies. Four articles concluded that chronic pain worsens cognitive function, whereas the others concluded the opposite that chronic pain does not worsen cognitive function. However, two studies with large sample sizes [25, 27] both found that chronic pain is related to cognitive impairment. Those articles reporting that pain does not worsen cognitive function had limitations in study design with small sample sizes, and other confounding factors.

**Table 4:** Studies focusing on population age over 65 years old.

Study sorted by author name	Number of participants (study group/control)	Pain type	Pain intensity	Study design	Results	Author-identified limitations
Murata et al. [16]	44 chronic musculoskeletal (MSK) pain group, 190 control group	Chronic MSK pain	Moderate (numeric rating scale, NRS>4)	Cross sectional	Chronic musculoskeletal pain may interfere with cognitive function elements (executive function, processing speed and semantic fluency) in community-dwelling older adults.	Cross-sectional study no MRI or fMRI Only on chronic MSK pain. Small difference in executive function between the study/control.
Scherder et al. [18]	19 patients in an early stage of probable AD, 20 older persons without dementia	Arthrosis/arthritis	Moderate	Cross sectional	In cognitively intact older persons, there is no significant correlation between specific cognitive functions and pain intensity/effect. In the Alzheimer's dementia group, there is a significant positive correlation.	The present findings can only be generalized to the older adults who do not suffer from a major depressive disorder
Schuler et al. [21]	55 chronic pain, 36 acute pain	Chronic and acute pain	Moderate	Cross-sectional	Report of pain intensity and improvement in the ADL measure was independent of cognitive status.	Small sample size
Terassi et al. [23]	187 elderly caregivers with chronic pain, 133 without chronic pain	Chronic pain	Moderate pain (39.0%). Severe pain (38.6%)	Cross sectional	No difference was found in cognitive performance among the elderly with chronic pain and those without chronic pain for any domain of the ACE-R instrument	No use of neuroimaging techniques or specific tests for memory, attention, and concentration. No information about the duration of chronic pain. No controlled variables (meds, depression, sleep disorders)
van der Leeuw et al. [25]	765 participants in Maintenance of Balance Independent Living Intellect and Zest Boston Study (MOBILIZE) study	Non-specific pain	Mild (brief pain inventory, BPI female 2.67; male 1.88)	Cross sectional	Elderly adults with more severe pain/pain interference have poorer performance on memory tests/executive functioning compared to elders with none or less pain	Cross-sectional relationships Individuals with significant cognitive impairment (MMSE < 18) were excluded from the MOBILIZE Boston cohort.
Weiner et al. [26]	160 cLBP, 163 pain-free patients	cLBP	Mild	Cross sectional	Osteoarthritis (OA) with cLBP demonstrated impaired NP performance as compared with pain-free OA	Cross-sectional design
Whitlock et al. [27]	1120 with persistent pain, 8945 controls	Persistent pain	Moderate to severe pain	Prospective cohort	Persistent pain was associated with accelerated memory decline and increased probability of dementia	Chronic pain patients had fewer evaluations d/t dropouts. Little info about source, nature, or treatment of pain. Potential confounding factors not measured
Zanocchi et al. [28]	105 patients	Chronic pain	N/A	Cross sectional	In institutionalized elderly, there was no correlation between chronic pain and cognitive function change	Small sample size. Difficulty removing confounding factor (age and sex). Difficulty assessing pain in patients with cognitive impairment. Many sample patients with moderate cognitive impairment.

 

Studies about whiplash associated disorder related pain 

Three studies focused on WAD-related pain, as it appears in Table 5. Two [7, 20] out of those three studies concluded that WAD-related pain worsens cognitive function. One study [10] reported that WAD-related pain is not associated with cognitive function but did not assess the long-term effects.

**Table 5:** Studies focusing on WAD: impact of pain on cognitive impairment. WAD, whiplash-associated disorder

Study sorted by author name	Number of participants (study group/control)	Pain intensity	Study design	Results	Author-identified limitations
Coppieters et al. [7]	16 chronic whiplash-associated disorder (WAD) patients, 21 fibromyalgia (FM) patients, and 22 pain-free volunteers	N/A	Case control study	Significant cognitive impairment, bottom-up sensitization, and decreased health-related QoL were demonstrated in patients with chronic WAD and FM compared to healthy controls.	Cross-sectional study
Ickmans et al. [10]	27 chronic WAD and 27 controls	Moderate (VAS Pre-exercise 57, Post-exercise 56)	Prospective cohort study	In the short term, post-exercise cognitive functioning does not worsen in people with chronic WAD	Longer-term effects of exercise (therapy) on cognitive functioning in people with chronic WAD need to be studied
Schmand et al. [20]	65 Whiplash non-malingering, 43 Whiplash malingering, 20 Closed head injury, 46 controls	Quebec classification criteria of whiplash-associated disorders, grades I-III	Cross sectional study	The cognitive complaints of non-malingering post-whiplash patients are likely a result of chronic pain, chronic fatigue, or depression.	N/A

Comparison of the results with other systematic reviews

A systematic review by de Aguiar et al. [47] concluded that persistent pain was not associated with cognitive impairment in geriatric populations. However, persistent pain was associated with cognitive decline in cases of follow-up length less than 4.5 years. Differences in inclusion and exclusion criteria exist between this systematic review and ours. In our review, transient changes or subjectively reported cognitive function were excluded. Headache was not included in our study due to its often intermittent nature.

Part 2: Impact of opioid pain medication on cognitive impairment

Demographic information

Demographic information is available in Table 6. Out of 18 reviewed studies, four studied geriatric populations over 65 years old. Five studies did not have a control group, but simply followed a cohort of patients across time. There was no significant male or female preponderance in the patient population.

**Table 6:** Demographic information: impact of opioids on cognitive impairment.

Study sorted by author name	Number of participants (study group; control)	Age (years old) (study group; control)	Gender (study group male/female; control group male/female)
Byas-Smith et al. [29]	Opioid Group 21; Nonopioid Group 11; Normal Group 50	47.7; 46.5; 42.6	10/11; 5/6; 23/27
Cherrier et al. [30]	Healthy, older (> 65 years) 36; middle-aged (35 to 55 years) adults 35	74.39; 48.42	16/20; 15/20
Dagtekin et al. [31]	Chronic noncancer pain patients treated with stable doses of transdermal buprenorphine 30; healthy volunteer controls 90	53; 53	19/11; 57/33
Dublin et al. [32]	0-10 TSD (total standardized doses) 1852;11-30 TSD 830; 31-90 TSD 476; 90+ TSD 276	74;75;75;76	0-10 TSD 759/1093; 11-30 TSD 340/490; 31-90 TSD 195/281; 90+ TSD 83/193
Gaertner et al. [33]	Oxycodone group 30; control group 90	55; 55	23/7; 69/21
Galski et al. [34]	Chronic Opioid Analgesic Therapy 16; cerebrally compromised patients 327 [Behind the wheel test (BTW) pass 162, BTW fail 165]	48.38; (45.87; 46.62)	N/A
Gianni et al. [35]	93 patients who received transdermal buprenorphine	79.7 (65-96)	24/69
Jamison et al. [36]	144 low back pain patients	46.3 (21-70)	87/57
Kurita et al. [37]	49	50.5	24/25
Menefee et al. [38]	23 patients taking less than a 15mg equivalent of oxycodone per day	47	6/17
Nilsen et al. [39]	Patients not using opioids 20; long-term codeine therapy 20; healthy controls 20	42.4; 43.2; 37.7	7/13; 10/10; 8/12
Panjabi et al. [40]	84	46.6 (19-65)	41/43
Richards et al. [41]	cLBP taking opioids 18; cLBP not taking opioids 22; control 20	60(28-81); 65(26-89); 57(24-76)	10/8; 12/10; 14/6
Sabatowski et al. [42]	21;90	50(34-65); 50(34-65)	18/12; 57/33
Schumacher et al. [43]	20; 19	54; 43	13/7; 12/7
Sjogren et al. [44]	40; 40	60(46-74); 59(49-78)	16/24; 11/29
Tassain et al. [45]	18; 10	46(18-65); 51.4(27-65)	10/8; 1/9
Won et al. [46]	Standing long-acting opioids 120; standing short acting opioids + nonopioids 693; standing nonopioids 1389; no analgesics 1467	81; 83.9; 83; 83.6	15/105; 77/616; 241/1128; 329/1138

Study design

Prospective cohort studies (14 out of 18 studies being reviewed) were most common, followed by two cross-sectional studies, one retrospective cohort study, and one randomized controlled trial.

Pain type, chronicity, and intensity

All reviewed studies focused on chronic pain unless otherwise specified. Mixed pain etiologies (musculoskeletal, visceral, and neuropathic) were most studied in nine articles. Two studies included patients with chronic low back pain (cLBP). Six studies did not report the type of pain. One study included only participants without any pain. Study subjects had moderate intensity of pain in 11 studies, and mild pain in two studies. The remaining five studies did not report pain intensity.

Opioids

Diverse opioid analgesics were employed in seven out of 18 reviewed studies. There was no consistency in the type of opioids, which caused difficulty in merging their findings. Oxycodone, morphine, transdermal fentanyl, and transdermal buprenorphine were used by subjects in two studies each. Codeine was used in one study, and there were two studies that did not present information about the type of opioid analgesics. Opioid doses also differed widely between studies. In most studies, there was very limited information about the duration of treatment and the other concomitantly used pain medications.

Cognitive tests 

**Figure 4:**
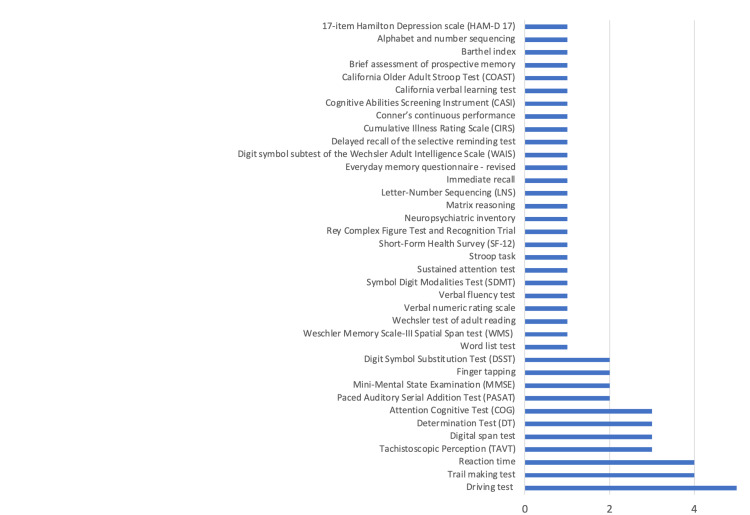
Numbers of studies using specific cognitive tests (number of participants): impact of opioids on cognitive impairment.

Figure 4 shows what type of cognitive tests were addressed in the studies. The driving function assessment test was most used to assess cognitive function, followed by the trail-making test and the reaction time test. Five articles assessed driving function. Three assessed driving function indirectly, with the German national recommendations test battery, which is a panel of three psychophysical tests consisting of an attention cognitive test, a determination test, and a tachistoscopic perception test. The tests that were commonly used in the articles about the impact of pain on cognitive impairment were used much less in this part of the review, such as the Stroop task. This lack of consistency limits the ability to aggregate the results.

Results

Fifteen out of 18 articles concluded that using opioids does not cause cognitive impairment. Two articles concluded that opioids do worsen cognitive function. One study [41] by Richards et al. concluded that opioid analgesics worsen attention but there was no statistically significant relationship between opioids and memory/executive function. 

Three studies [30, 41, 44] that concluded that the use of opioids impairs cognitive function had limitations in study design, including but not limited to a small sample size, as described in Table 7. And none of them addressed driving function which was most used to assess cognition in other studies. The study by Cherrier et al. [30] has a limitation of study design, as the subjects who did not have chronic or significant daily pain were assessed shortly after a dose of opioid. In the study by Richards et al. [41], subjects used a relatively small dose of opioid. The study by Sjogren et al. [44] focused on subjects with mild pain intensity; potentially confounding factors such as anxiety or depression were not well addressed.

**Table 7:** Studies stating that opioids worsen cognitive function: impact of opioids on cognitive impairment.

Study sorted by author name	Number of participants: study group; control	Pain type	Pain intensity	Type of opioids	Study type	Results	Author-identified limitations
Cherrier et al. [30]	Healthy, older (> 65 years) 36; middle-aged (35 to 55 years) adults 35	N/A (Patients who were not suffering from chronic or significant daily pain were included in the study)		Immediate release oxycodone	Randomized controlled trial	Significant declines in simple and sustained attention, working memory and verbal memory were observed at one-hour post dose compared to baseline with a trend toward a return to baseline by five hours post dose. No difference between healthy older adults and middle age adults.	Healthy, pain-free subjects. Placebo practice effect
Richards et al. [41]	cLBP taking opioids 18; cLBP not taking opioids 22; control 20	cLBP	Moderate (6/10)	N/A	Cross sectional	Patients receiving opioid analgesics performed significantly (p<0.05) worse in attention than those patients not taking opioids. Patient groups did not show a significant difference in memory and executive function. Patients with cLBP performed significantly worse in areas of attention and executive working memory than the healthy control participants. (p<0.05)	No data for some cytokine concentrations. Opioid status determined from medical records. Heterogeneous opioid doses.
Sjogren et al. [44]	40 on opioids; 40 healthy control	Chronic Nonmalignant Pain	Mild (study group subjective assessment of sedation intensity, SVAS 27/100, subjective assessment of pain intensity, PVAS 39/100; control group SVAS 5/100, PVAS 0/100)	Sustained-release morphine, methadone, ketobemidone, buprenorphine, tramadol	Prospective cohort	The patients who received long-term oral opioid therapy for chronic nonmalignant pain performed statistically significantly poorer than the controls in all the cognitive tests.	Not opioid naive. Not controlled for other pain meds. Confounding factor – 50% anxiety, 38% depression.

Studies focusing on ages over 65 years old 

As it is described in Table 8, three [32, 35, 46] of four studies on the geriatric population concluded that using opioid analgesics does not necessarily worsen cognitive function. One study [30] concluded that opioids worsen cognitive function. Studies with large sample sizes [27, 41] showed consistent results that using opioids does not worsen cognitive function.

**Table 8:** Studies focusing on age group over 65 years old: impact of opioids on cognitive impairment.

Study sorted by author name	Number of participants (study group; control)	Control	Pain type	Pain intensity	Type of opioids	Study type	Results	Author-identified limitations
Cherrier et al. [30]	Healthy, older (> 65 years) 36; middle-aged (35 to 55 years) adults 35	Y	N/A (Patients who were not suffering from chronic or significant daily pain were included in the study)		Immediate release oxycodone	Randomized controlled trial	Significant declines in simple and sustained attention, working memory and verbal memory were observed at 1 h post dose compared to baseline with a trend toward return to baseline by 5 h post dose. No difference between healthy older adults and middle age adults.	Healthy, pain-free subjects only. Placebo practice effect
Dublin et al. [32]	0-10 TSD (total standardized doses) 1852;11-30 TSD 830; 31-90 TSD 476; 90+ TSD 276	Y	N/A		Codeine, oxycodone, hydrocodone	Prospective cohort study	Heavier opioid use was not associated with more rapid cognitive decline. For cumulative opioid use, the hazard ratios (HRs) for dementia were 1.06 for 11 to 30 TSDs (total standardized doses), 0.88 for 31 to 90 TSDs, and 1.29 for 91 or more TSDs, versus 0 to 10 TSDs.	Confounding factors (no info about pain chronicity, severity, etc.) Misclassification of NSAID use – over the counter (OTC) non-steroidal anti-inflammatory drugs (NSAIDS) were available during the study. Mostly Caucasian
Gianni et al. [35]	93 patients who received transdermal buprenorphine	N	Chronic noncancer pain, mixed types	Moderate to severe	Buprenorphine transdermal therapeutic system (TDS)	Prospective observational one group	Use of Buprenorphine TDS showed an improvement in mood and a partial resumption of activities, with no influence on cognitive and behavioral ability	N/A
Won et al. [46]	Long-acting opioids (LAOs) 120; short-acting opioids (SAOs) + nonopioids 693; nonopioids 1389; no analgesics 1467	Y	Mixed type of pains	Mild to moderate pain	Long-acting opioids, short-acting opioids	Prospective cohort study	There were no changes in cognitive status or mood status, or increased risk of depression with use of any analgesics, including opioids. There was a trend toward a lower risk of falls with use of any analgesics (HR 0.87).	Severely restricted sample (nursing home residents without dementia). The study design contributing to low rates of adverse events. Small number of samples on long acting opioids (LAOs) or short acting opioids (SAOs).

Driving test results as a marker of cognitive/executive function

Five studies directly assessed driving function as described in Table 9.

**Table 9:** Studies employing driving tests to test cognitive function: impact of opioids on cognitive impairment.

Study sorted by author name	No of participants: study group; control	Control	Pain type	Pain intensity	Opioids	Study type	Results	Author-identified limitations
Byas-Smith et al. [29]	Opioid Group 21; Nonopioid Group 11; Normal Group 50	Y	Chronic persistent daily pain	N/A	Mixed	Retrospective cohort	No signiﬁcant differences were observed among groups in driving performance (in the community, on the obstacle course, on the Test of Variables of Attention).	Convenience sample aware of being evaluated. Short duration of the driving test.
Galski et al. [34]	Chronic Opioid Analgesic Therapy (COAT) 16; cerebrally compromised patients (CCOMP) 327	Y	Non-malignant pain	Mild (mean NRS 3.65) after COAT	N/A	Prospective cohort	COAT did not significantly impair the perception, cognition, coordination, and behavior measured in off-road tests that have been regarded as requisite for on-road driving. (Behind the wheel test pass 162, BTW fail 165)	No standardized, valid, and reliable procedures for driving evaluations. Limited sample of COAT patients. No controls who are healthy or nonopioid using pain patients. Heterogeneity of pain etiologies. Confounding factors not addressed.
Menefee et al. [38]	23 patients taking less than a 15 mg equivalent of oxycodone per day	N	Mixed type of pains	Moderate	Transdermal fentanyl	Prospective observational one group	The addition of transdermal fentanyl to a no opioids treatment regimen or small amounts of opiates for chronic nonmalignant pain patients does not impair driving performances, reaction times, cognition, or balance.	Small sample size. Only driving simulation. No on-the-road test. Does not address the effects of transdermal fentanyl immediately after fentanyl. Fentanyl < 75 microgram/hour. No consideration on individual differences.
Nilsen et al. [39]	Chronic pain without (W/O) opioids 20; long-term codeine 20; healthy controls 20	Y	Mixed type of pains	Moderate	Codeine	Prospective cohort	Codeine does not impair driving-related abilities over and above what is associated with chronic pain per se.	Relatively small sample size.
Schumacher et al. [43]	20 chronic non cancer pain (CNCP) taking opioids; 19 healthy control	Y	Mixed type of pains	N/A	Fentanyl, buprenorphine, oxycodone, hydromorphone, morphine	Prospective cohort	Driving performance of CNCP patients did not significantly differ from that of controls, when performance of controls with a blood alcohol concentration of 0.5 g/L was used as a reference.	Patients treated with different analgesics at various dosages were enrolled. Small sample size.

Three studies indirectly assessed driving function using the German national recommendations test battery, as shown in Table 10.

**Table 10:** Studies indirectly assessing driving ability (German test battery) to assess cognitive function: impact of opioids on cognitive impairment.

Study sorted by author name	Number of participants (study group; control)	Control group	Pain type	Pain intensity	Type of opioids	Study type	Results	Author-identified limitations
Dagtekin et al. [31]	Chronic pain patients with transdermal buprenorphine 30; healthy volunteer controls 90	Y	Lower back pain, neuropathic pain, pain from miscellaneous diseases	Moderate	Transdermal buprenorphine	Prospective cohort	German national recommendations test battery Driving function of patients receiving transdermal buprenorphine were shown to be non-inferior to the control group	Individual variability of test results
Gaertner et al. [33]	Oxycodone group 30; control group 90	Y	Lower back pain, neuropathic pain, miscellaneous diseases	Moderate	Controlled release oxycodone (CRO)	Prospective cohort	German national recommendations test battery. Driving ability of patients receiving controlled-release oxycodone did not differ significantly compared to the age-independent control group.	Bi-exponential pharmacokinetic of CRO. Fast drug release might imitate an effect close to immediate release opioids. Individual variability
Sabatowski et al. [42]	21 patients with transdermal fentanyl; 90 healthy control	Y	Mixed type of pains	Mild	Transdermal fentanyl	Prospective cohort	German national recommendations test battery. Stable doses of transdermal fentanyl for the treatment of chronic non-cancer pain are not associated with significant impairments in psychomotor and cognitive performance (p<0.05)	30% of the patients in the study did take additional unreported drugs. Confounding factors not measured, such as mood disorders.

The sample sizes of studies using driving function as an indicator for cognitive function were relatively small, and the information about pain type and intensity was limited. There was no consistency in the type of opioids studied. All articles assessing driving function concluded that there is no significant driving function decline attributable to opioid use.

Comparison of the results with other systematic reviews

Three other systematic reviews were consistent with our findings. A systematic review by Allegri et al. [48] investigated the neuropsychological effects of long-term use of opioids in patients with chronic noncancer pain. It concluded that opioids reduce attention, but there was no statistically significant difference noted in the other areas of cognitive function. Another systematic review by Ferreira et al. [49] assessed the impact of opioids on driving skills. They could not identify impaired simulated driving performance in subjects taking regularly scheduled opioids for symptom control. Pask et al. [50] concluded in their systematic review that in the 10 studies that met their inclusion criteria, six showed no consistent effect, and four indicated impairment of a range of cognitive function in patients receiving higher doses of opioids.

## Conclusions

Discussion

This study is one of few studies reviewing the impact on cognitive impairment of both pain and opioid pain medications. We found that opioid analgesics may impair cognitive function, but undertreated pain impairs cognitive function more. However, this systematic review has limitations.

Heterogenous studies were reviewed. Type of pain, opioids, and cognitive tests differ substantially between studies. This heterogeneity limited our ability to merge the findings of diverse reports. There were many cross-sectional studies and prospective observational cohort studies. Only one randomized controlled trial was identified in our review. Subgroup analyses were limited by the small number of the articles.

In the future, more studies are needed that focus on the over 65 years old population. Better quality study designs such as randomized controlled trials or well-designed cohort studies will provide a higher level of evidence. Further validation and uniform application of tools to test cognitive function will be required. Future studies should have suitably large sample sizes, and adjust for important confounding factors, such as mood disorder, level of education/intelligence, and underlying cognitive impairment, among others. 

Conclusion and recommendation

In summary, evidence supports that chronic pain impairs cognitive function. In contrast, our identified studies found that using opioids does not significantly worsen cognition, including measures of executive function, memory, or driving ability.

Based on the present systematic reviews, we conclude that concerns over cognitive impairment by opioids should not prevent the treatment of moderate to severe pain and that untreated pain can impair cognitive function significantly. Careful use of opioids for moderate to severe pain does not necessarily worsen executive function, memory, or driving ability. However, our results cannot be generalized as they are derived from heterogeneous studies and cohorts. An open question remains whether nonsedating, nonopioid analgesics might become analgesics of choice to control pain without impairing cognition.
